# Gaming the Mind – Improving Access to Recreational Gaming Activities Within an Inpatient CAMHS Unit

**DOI:** 10.1192/bjo.2024.408

**Published:** 2024-08-01

**Authors:** Adam Osman, Hamilton Morrin

**Affiliations:** South London & Maudsley NHS Trust, London, United Kingdom

## Abstract

**Aims:**

1. To assess the current video gaming provisions on an inpatient CAMHS ward.
2. To gather feedback from young people on the ward on the current provisions and gather suggestions for improvements.
3. To implement any suggestions of improvements.
4. To organise regular group gaming sessions for groups and to allow access for individual use.
5. To gather feedback following implementation on how to improve further.

**Methods:**

Using surveys, we gathered feedback on attitudes to recreational gaming, interest in gaming social events, preference for individual gaming and on thoughts of the current gaming facilities in a London inpatient CAMHS unit, from a cohort of young people admitted to the ward.

Based on this feedback we sourced funding for a Nintendo Switch from Gaming the Mind Charity and purchased a Nintendo Switch for the Unit along with preferred games.

Regular group gaming sessions were integrated into the ward activity schedule. Additionally, access for individual use was also facilitated.

Further feedback on implementation was gathered from the young people.

**Results:**

Based on initial survey feedback, a majority of young people on the ward indicated that they enjoyed gaming and that it was a good way to “*have fun, relax or socialise”.*

Feedback suggested the current provisions on the ward (consoles and games) were outdated and not used frequently.

Nintendo Switch received most suggestions for best new addition to the ward.

Game suggestions included: Mario Kart 8 deluxe, Wii sports, Splatoon 3, Mario super smash bros and Minecraft.

After implementation of social gaming events into ward timetable, follow up feedback was positive, suggesting that the majority of young people who engaged in recreational gaming activities on the ward benefited from this. Feedback suggested it was beneficial in terms of mood, socialising, and as a distraction from difficult emotions.

**Conclusion:**

Improving access to recreational video gaming consoles and games within inpatient settings is a valuable way to offer activities to improve mood and social interactions between young people in an inpatient CAMHS setting. Follow up research into efficacy would be of benefit.

